# Argonaute proteins: potential biomarkers for human colon cancer

**DOI:** 10.1186/1471-2407-10-38

**Published:** 2010-02-10

**Authors:** Lan Li, Chaohui Yu, Hengjun Gao, Youming Li

**Affiliations:** 1 Department of Gastroenterology, the First Affiliated Hospital, College of Medicine, Zhejiang University, Hangzhou, 310003, China; 2 Institute of Digestive Disease, Tongji Hospital affiliated to Tongji University, Shanghai, 200065, China

## Abstract

**Background:**

Although Argonaute proteins are considered to play important roles in stem cell self-renewal, RNA interference (RNAi) and translational regulation, relatively little is known about their functions in human disease. In this study, we investigated the expression of eight members of human Argonaute family in colon cancer and identified their potential roles in tumor development and progression.

**Methods:**

Antibodies against human Argonaute proteins were prepared by immunizing rabbits with synthetic peptides derived from the sequences of Argonaute members. Then we constructed a tissue microarray containing 75 specimens from colon cancer and 75 specimens from adjacent non-cancer tissue, and assayed eight different proteins (EIF2C1, EIF2C2, EIF2C3, EIF2C4, PIWIL1, PIWIL2, PIWIL3 and PIWIL4) by immunohistochemistry on consecutive formalin-fixed tissue microarray sections.

**Results:**

The expression of EIF2C1-4 and PIWIL1-4 was significantly higher in tumorous tissue than in adjacent tissue. Notably, a significant correlation was observed between the positive expression of EIF2C2, EIF2C3, EIF2C4, PIWIL4 and the presence of distant metastasis. Logistic regression analysis revealed that an increased expression of EIF2C1 and PIWIL2 was significantly associated with occurrence of colon cancer tissue compared with non-cancer tissue.

**Conclusions:**

Argonaute proteins are overexpressed in colon cancer relative to adjacent non-cancer tissue. The expression of EIF2C2-4 and PIWIL4 appears increased in advanced tumors with distant metastasis, suggesting it may promote tumor invasion. Furthermore, EIF2C1 and PIWIL2 might represent novel colon cancer markers with early diagnostic significance.

## Background

Colon cancer is one of the most frequent and lethal malignancies worldwide, and the 5-year survival rate is less than 50% [1,2]. Given the high level in incidence rate and mortality rate of colon cancer, it would be important to better understand the biological basis of tumor development and progression, to develop markers for assessing onset or prediction of therapy outcome, as well as to identify targets for the development of novel therapies. Colon cancer may be considered the final step of a progressive imbalance between mucosal cell proliferation and apoptosis due to the activation of oncogenes and the inactivation of tumor suppressor genes [3-5]. The evaluation of the clinical utility of each of these genes would require multiple consecutive experiments with hundreds of tumor specimens. This would be both time-consuming as well as impractical for more than a handful of genes. Microarray technology provides a new and promising tool that allows the detection of a large variety of parameters simultaneously, and will be of importance in the fight against colon cancer.

Argonaute proteins are present in all RNA-induced silencing complexes (RISC) reported to date and are now the best defined protein component of the RNA interference (RNAi) machinery [6]. Humans have eight Argonaute-like proteins, four of which fall into the eIF2C/AGO subfamily (EIF2C1/hAGO1, EIF2C2/hAGO2, EIF2C3/hAGO3, and EIF2C4/hAGO4) while the remainders are the PIWI subfamily (PIWIL1/HIWI, PIWIL2/HILI, PIWIL3, and PIWIL4/HIWI2) [7]. The AGO subfamily is present in animals, plants, and fission yeast. Proteins of this subfamily use small interfering RNAs (siRNAs) and/or microRNAs (miRNAs) as sequence specific guides in both transcriptional and posttranscriptional silencing mechanisms [8]. It is postulated that eIF2C proteins might have regulatory functions in cancer stem cell self-renewal through the RNA-mediated gene silencing mechanism as a component of RISC. In contrast to the AGO subfamily, the PIWI subfamily has been identified only in animals. The *PIWI *subfamily genes are expressed mainly in germ cells, whereas *AGO *subfamily genes are ubiquitously expressed. Consistent with their expression patterns, PIWI proteins may participate in germ cell proliferation and their overexpression may cause germ cell malignancy development [9]. Although Argonaute proteins are considered to play important roles in RNA interference, stem cell self-renewal and translational regulation, relatively little is known about their functions in human disease. In the present study, we constructed a tissue microarray containing 150 specimens from adjacent non-cancer tissue and colon cancer tissue and assayed the expression of eight members of human Argonaute family by immunohistochemistry on consecutive formalin-fixed tissue microarray sections. The aim was to obtain a comprehensive survey of the expression of Argonaute proteins in colon cancer and identify their potential roles in tumor development and progression.

## Methods

### Patients and colonic specimens

We selected 75 patients with colon cancer who underwent surgery at hospitals that cooperated with Shanghai Outdo Biotech Co., Ltd. during 2005-2007. They were 38 men and 37 women, ranging from 25 to 85 years of age (median: 57 years). Clinicopathaological characteristics of the research subjects are shown in Table 1. Paraffin-embedded diagnostic tumor biopsy specimens and their adjacent non-tumor specimens (≤ 1.5 cm away from the tumor) were collected before any treatment.

**Table 1:** Clinicopathaological characteristics of colon cancer cases

Characteristic	No. (n = 75)	%
Age, years		
<60	30	40
≥60	45	60
Sex		
Female	37	49.3
Male	38	50.7
Duke's stage		
A	9	12
B	31	41.3
C	28	37.3
D	7	9.3
Histologic type		
Adenocarcinoma	72	96
Mucinous carcinoma	2	2.7
Signet-ring cell carcinoma	1	1.3
Histologic grade		
I	8	10.7
II	54	72
III	13	17.3
Lymph nodes metastasis		
Absence	44	58.7
Presence	31	41.3
Distant metastasis		
Absence	68	90.7
Presence	7	9.3

Patients were only included in the study if they had provided written consent to participate in the study after receiving oral and written information regarding its course and purpose. Approval for the study was received from the Ethics Committee of the host institution.

### Preparation of antibodies against Argonaute proteins

The optimal peptide immunogens for preparation of antibodies against human Argonaute proteins were selected by an in-house peptide selection database called Antibody Designer, and then synthesized and fractionated by C-Strong Corporation (Shanghai, China). The amino acid sequences for each peptide antigen are collected in Table 2. The synthetic peptide antigens (KLH-coupled) were used to raise polyclonal antibodies in rabbits as described by Pastor-Navarro N et al. [10]. Evolution of the antibody titer was controlled by measuring the binding of serial dilutions of the antisera to microtiter plates coated with peptide antigen. Eight weeks after the first injection, sera were collected and used to prepare purified antibodies. The IgG solution was fractionated from the rabbit antisera by precipitation with 40% saturated ammonium sulphate and then affinity-purified on peptide affinity columns. The flow-through was collected and stored at -20°C for the use of ELISA, Western blot, and immunohistochemistry analyses.

**Table 2:** Amino acid sequences of immunogens for preparation of antibodies against human Argonaute proteins

	Sequences	AA number
EIF2C1	^144^ALVSGQIPVPLESV^157^-C	14
EIF2C2	^137^CVSLQALHDALSGR^150^-C	14
EIF2C3	^144^TLPEPLELDK^153^-C	10
EIF2C4	^632^SQELLYSQEVIQ^643^-C	12
PIWIL1	^440^DWGLSFDSNLLSFSGR^455^-C	16
PIWIL2	^345^DPTSAMVLQQHR^356^-C	12
PIWIL3	^78^EPGPEAGLHTAPL^90^-C	13
PIWIL4	^434^WGLHFGSQISLTGR^447^-C	14

AA: amino acid.

### Tissue microarray construction

The colonic tissue microarray was constructed as described previously [11]. Briefly, a tissue arraying instrument (Beecher Instruments, Silver Spring, MD) was used to create holes in a recipient paraffin block and to acquire tissue cores from the donor block by a thin-walled needle with an inner diameter of 1.5 mm, held in an X-Y precision guide. The cylindrical sample from the selected region in the donor block was extruded directly into the recipient block with defined array coordinates. After construction of the array block, multiple 5-μm sections were cut with a microtome and placed on polylysine-coated slides. The tissue array block contained 150 samples, including 75 tumorous specimens and 75 adjacent non-tumorous specimens.

### Immunohistochemistry

Immunohistochemical staining was performed using two-step method. The sections were deparaffinized and rehydrated. Antigen retrieval was performed by autoclaving the slides in 10 mM citric acid buffer. Antibodies against Argonaute proteins (dilution of 1:100), and goat anti-rabbit immunoglobulin/HRP from DAKO were selected as primary antibody and secondary antibody. Reaction products were visualized with diaminobenzidine as the chromogen and finally counterstained with haematoxylin.

The immunohistochemical expression of each marker was examined by light microscopy through calculating 1000 cells per 5 sights and evaluating the average number. The percentage of positive cells, as the extent of immunostaining, was quantified under microscope and classified into five groups. 0: < 5% positive cells; 1: 5% to 24% positive cells; 2: 25% to 49% positive cells; 3: 50% to 74% positive cells and 4: ≥ 75% positive cells. Intensity was scored as 0 for absence of staining, 1 for weak, 2 for moderate, and 3 for strong staining. The score of the intensity plus the percentage of positive staining was used to define expression levels. 0-1: negative; 2-3: little positive (+1); 4-5: moderately positive (+2); 6-7: strongly positive (+3).

### Statistical analysis

The differential expression of Argonaute proteins between tumorous tissue and non-tumorous tissue was determined by Mann-Whitney U-test. Relationships between clinicopathological and molecular parameters were statistically analyzed using Spearman's rank correlation coefficient. The influence of each variable on histotype of colonic lesion was assessed by logistic regression analysis. A value of *P *< 0.05 was considered to be statistically significant.

## Results

### Production and validation of antibodies against Argonauteproteins

**Figure 1:**
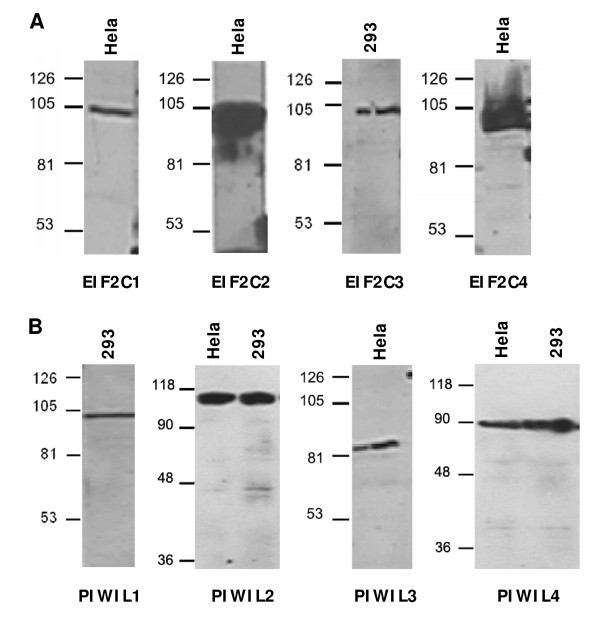
**Western blot analysis of rabbit polyclonal antibodies against Argonaute proteins in Hela or 293 cell lines**. (**A**) Total protein extracted from Hela or 293 cells was subjected to Western blot with polyclonal antibodies against EIF2C1-4 proteins. There were the bands at expected molecule mass corresponding to each member of AGO subfamily in the films. (**B**) Total protein extracted from Hela or 293 cells was subjected to Western blot with polyclonal antibodies against PIWIL1-4 proteins. There were the bands at expected molecule mass corresponding to each member of PIWI subfamily in the films.

An in-house peptide selection database called Antibody Designer was used to select optimal peptide immunogens for production of antibodies against human Argonaute proteins. For each protein, a synthetic peptide derived from the sequence of Argonaute member was conjugated to KLH for immunization. All peptides showed strong immune responses after 4 immunizations in rabbits, and antisera exhibited high titers when tested by ELISA using immobilized peptides on 96-well microtiter plates. Antibodies from individual rabbits were purified separately by peptide affinity chromatography. Typically 2-20 milligrams of purified antibodies were obtained for each peptide. SDS-PAGE analysis showed that the molecular mass of purified antibodies was 50 kDa, which corresponded to the molecular mass of rabbit IgG. Total protein extracted from either Hela or 293 cells was used for immunoblotting with purified antibodies. Western blot analysis showed that these purified antibodies recognized the bands at expected molecule mass corresponding to each Argonaute member, respectively (Figure 1). In contrast, no band was detected with preimmune rabbit serum. Furthermore, immunohistochemical analysis showed predominantly cytoplasmic staining in most tumorous tissues but very weak or absent staining in normal human tissues.

### Argonaute proteins expression in adjacent non-tumorous tissue and tumorous tissue

**Figure 2:**
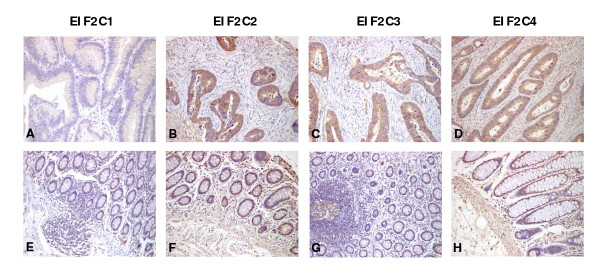
**Immunohistochemical expression of EIF2C1, EIF2C2, EIF2C3 and EIF2C4 in colon cancer and adjacent non-cancer tissue**. In colon cancer tissue EIF2C1-4 expression was often stronger in the cytoplasm compared with adjacent non-cancer tissue (**A, B, C, D**). Adjacent non-cancer tissue is detected with very weak EIF2C1-4 expression in the cytoplasm (**E, F, G, H**). Magnifications: ×200.

**Figure 3:**
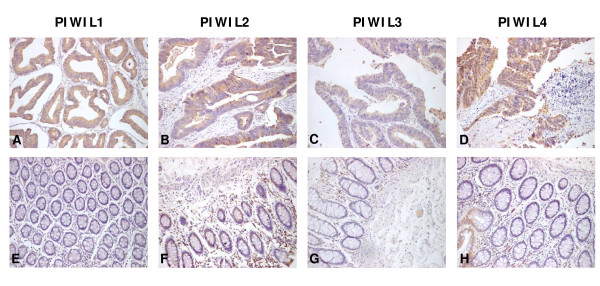
**Immunohistochemical expression of PIWIL1, PIWIL2, PIWIL3 and PIWIL4 in colon cancer and adjacent non-cancer tissue**. In colon cancer tissue PIWIL1-4 expression was often stronger in the cytoplasm compared with adjacent non-cancer tissue (**A, B, C, D**). Adjacent non-cancer tissue is detected with very weak PIWIL1-4 expression in the cytoplasm (**E, F, G, H**). Magnifications: ×200.

By use of a large tissue microarray (150 cores) we investigated the protein expression of EIF2C1-4 and PIWIL1-4 in colon cancer specimens and adjacent non-tumorous tissue. The tumorous or non-tumorous mucosa-specific staining was semi-quantitatively scored by the intensity and the percentage of positive staining. As shown in Figure 2 and 3, Argonaute proteins expression was detected mainly in cytoplasm of malignant cells. The positive expression of EIF2C1 (*P *< 0.001), EIF2C2 (*P *< 0.001), EIF2C3 (*P *< 0.001), EIF2C4 (*P *< 0.001), PIWIL1 (*P *< 0.001), PIWIL2 (*P *< 0.001), PIWIL3 (*P *< 0.001) and PIWIL4 (*P *< 0.001) in tumorous tissue was significantly higher than in adjacent non-tumorous tissue. Images of representative immunostaining are presented in Figure 2 and 3. The results are shown in Table 3.

**Table 3:** EIF2C1-4 and PIWIL1-4 expression in adjacent non-tumorous tissue and tumorous tissue

Marker	Histotype^a^	Expression levels (number)			
		
		-	+1	+2	+3
EIF2C1	T	1	11	48	15
	N	31	29	14	1
EIF2C2	T	1	0	30	44
	N	5	32	25	13
EIF2C3	T	1	1	49	24
	N	10	35	15	15
EIF2C4	T	0	1	38	36
	N	14	39	10	12
PIWIL-1	T	1	4	56	14
	N	16	36	19	4
PIWIL-2	T	1	1	46	27
	N	23	30	20	2
PIWIL-3	T	3	16	47	9
	N	33	31	10	1
PIWIL-4	T	1	4	49	21
	N	23	34	11	7

^a^T: Tumorous tissue; N: Non-tumorous tissue.

### Relationship between the expression of Argonaute proteins and clinicopathological parameters

On the basis of the above results in the colon cancer specimens, it would be interesting to evaluate the correlation between the expression of Argonaute proteins and clinicopathological parameters in colon cancer. As shown in Table 4, there were no statistical differences among each protein expression and age, sex, histological grade, lymph node status or Duke's stage. The positive expression of EIF2C2 (r = 0.268, *P *< 0.05), EIF2C3 (r = 0.269, *P *< 0.05), EIF2C4 (r = 0.242, *P *< 0.05) and PIWIL4 (r = 0.301, *P *< 0.01) in colon cancer was associated with the presence of distant metastasis.

**Table 4:** Relationship between the expression of Argonaute proteins and clinicopathological parameters

Marker	Correlation coefficient (r)					
	
	Sex	Age	Duke's stage	Histologic grade	LN status	Distant metastasis
EIF2C1	0.074	-0.082	-0.121	-0.048	-0.001	0.130
EIF2C2	0.080	0.078	0.022	0.019	-0.163	0.268^a^
EIF2C3	0.100	-0.053	0.209	0.200	0.091	0.269^a^
EIF2C4	0.026	0.010	0.090	0.089	-0.116	0.242^a^
PIWIL-1	0.038	0.149	0.100	-0.135	-0.024	0.205
PIWIL-2	0.123	-0.093	-0.126	-0.025	-0.087	-0.004
PIWIL-3	-0.008	0.214	0.026	-0.003	-0.097	0.146
PIWIL-4	0.014	-0.033	0.064	-0.146	-0.111	0.301^b^

^a^*P *< 0.05, ^b^*P *< 0.01, LN: lymph node.

### Logistic regression analysis of influence on histotype ofcolonic lesion

A stepwise forward logistic regression was used to assess the effects of EIF2C1-4 and PIWIL1-4 on histotype of colonic lesion (whether the histotype of colonic lesion was tumorous tissue or adjacent non-tumorous tissue). Logistic regression analysis revealed that an increased expression of EIF2C1 and PIWIL2 was significantly associated with occurrence of colon cancer tissue (OR = 3.071, *P *= 0.005 and OR = 7.392, *P *< 0.001, respectively) (Data are shown in Table 5). However, the rest of human Argonaute proteins had no significant effects on histotype of colonic lesion.

**Table 5:** Logistic regression analysis of the effect of Argonaute proteins on histotype of colonic lesion

Variable	Expression levels	**No**.	% Tumorous tissue	Wald χ^2^	*P*	OR	95% CI
EIF2C1	-	32	3.13				
	+1	40	27.5				
	+2	62	77.42				
	+3	16	93.75	7.723	0.005	3.071	1.392-6.777
PIWIL2	-	24	4.17				
	+1	31	3.23				
	+2	66	69.70				
	+3	29	93.10	14.894	0.000	7.392	2.676-20.414

## Discussion

Here, we performed a first systematic expression analysis of human Argonaute proteins on a cohort of 75 Chinese colon cancer specimens and subsequently identified potential roles for Argonaute proteins in the development and progression of colon cancer. Because of the lack of commercially available antibodies against Argonaute proteins, we prepared eight rabbit polyclonal antibodies that recognized human Argonaute proteins efficiently. With the antibodies, we detected the tissue distribution of EIF2C1-4 and PIWIL1-4 by immunohistochemistry on tissue microarray.

Although several human Argonaute proteins have been identified, relatively little is known about their functions in human disease. AGO subfamily members are components essential for siRNA-mediated gene silencing in mammalian cells and involved in the effecter step of mammalian RNAi [12,13]. Recent studies demonstrate that the human *EIF2C1 *gene is located on the short arm of chromosome 1 in the region 1p34-p35. This genomic region is frequently lost in human cancers such as Wilms tumors, neuroblastoma, and carcinomas of the breast, liver, and colon [14]. The human *EIF2C1 *gene is ubiquitously expressed at low to medium levels, and *EIF2C1 *expression was found to be elevated in Wilms tumors that lacked functional copies of the Wilms tumor suppressor gene *WT1 *[14]. Together, these findings could make human *EIF2C1 *an interesting candidate gene for potential involvement in neoplastic development. In our study, it should be noted that positive reaction to each AGO in colon cancer tissue was significantly higher than that in adjacent non-cancerous tissues. The relationship of AGO subfamily with colon cancer has not been completely elucidated. Perhaps through RNAi-related pathways or possibly also through distinct mechanisms, AGO subfamily members have an important role in the progression of colon cancer.

As a subfamily of Argonaute proteins, PIWI proteins are expressed in the germline and in somatic cells as well [15]. Four PIWI-like proteins have been identified in Homo sapiens. Unlike AGO subfamily proteins, PIWI subfamily proteins do not associate with siRNAs and/or miRNAs, or they do so to a lesser extent [9]. Recently, it has become clear that PIWI subfamily proteins bind to a third class of small RNAs called PIWI-interacting RNAs (piRNAs) [16-19]. piRNAs and PIWIs appear to be involved in the epigenetic control of gene expression, the control of mRNA stability, transposon silencing and translation regulation [6,20,21]. Elevated expression of PIWI subfamily has been reported in several human tumor entities. First, PIWIL1 expression has been analysed in male germline cells, showing that mRNA levels of *PIWIL1 *was upregulated in the occurrence of seminomas, that is, a type of testicular germ cell tumors [22]. Liu et al. [23] showed that the percentage of cells that expressed PIWIL1 increased from 10% in normal gastric tissues to 76% in gastric cancer. In addition, expression of the PIWIL2 protein was also found in different tumors examined, including prostate, breast, pancreatic, gastrointestinal, ovarian and endometrial cancer of human and in breast tumors, rhabdomyosarcoma and medulloblastoma of mouse [24]. Our investigation showed that the positive rate of PIWI protein expression in colon cancer tissue was remarkably higher than that in non-cancer tissue. These results confirm the notion derived from in vitro experiments that PIWI members might be induced by oncogenic event [22-26]. A possible involvement of PIWI subfamily in the development and progression of colon cancer is proposed.

In order to identify markers associated with clinicopathological characteristics of colon cancer patients, the relationship between sex, age, histological grade, metastasis, Duke's classification, and protein expression needed additional research. Previous studies rarely considered the clinical meaning of the presence of AGOs and PIWIs in colon cancer. In the present study, we found the positive correlation of EIF2C2, EIF2C3, EIF2C4 and PIWIL4 with tumor distant metastasis. It is suggested that EIF2C2-4 and PIWIL4 are associated with tumor progression to advanced stage and may promote tumor invasion. However, there were no statistical differences for each protein expression among sex, age, histological grade and Duke's stage. Larger studies with a higher number of samples are necessary to confirm these results and to identify the prognostic value of Argonaute proteins in colon cancer.

A goal of this project is to identify biomarkers occurring in colonic carcinogenesis and contributing to colon cancer development and progression. Of the EIF2C1-4 and PIWIL1-4 analyzed by logistic regression, we observed that an increased expression of EIF2C1 and PIWIL2 was significantly associated with occurrence of colon cancer tissue compared with non-cancer tissue. This is of potential clinical importance for early diagnosis. The question that remains to be discussed is how Argonaute proteins play an important role in colonic carcinogenesis. It has been postulated that mutations or overexpression of several Argonaute proteins might cause cancer stem cells to unlimited self-renewal and aberrant differentiation, resulting in a heterogeneous population of cells [27,28]. According to the cancer stem cells hypothesis, EIF2C1 and PIWIL2 proteins might play a role in the balance between colonic cancer stem cells self-renewal and division in association with small RNAs' pathway. A disturbance in this balance may have strong impact on neoplastic development [26]. Coincidentally, several previous studies have reported that the gene silencing of PIWIs by RNAi or antisense technology inhibited the growth of cancer cells and induced cell cycle arrest in G2/M phase in human gastric cancer and seminomas [23,24], which supported the issue that overexpression of Argonaute members was associated with proliferation and apoptosis of cancer stem cell.

The interaction of Argonaute proteins with small RNAs or other part of RISC involved in carcinogenesis has not been completely elucidated. Gene specific translational control induced by some miRNA species has been reported to have an effect on cancer development [29]. As a component of RISC, Argonaute proteins bind to miRNAs or piRNAs, and aberrant regulation of these small RNAs by Argonaute proteins might induce the malignant phenotype of cells. SND1, also reported to be a component of RISC, is overexpressed in human colon cancer tissues, even in early-stage lesions [30-32]. The relationship of SND1 with Argonaute proteins was still unclear. Paukku et al. [30] reported that the effect of SND1 through 3'-untranslated region of angiotensin II type 1 receptor was independent of EIF2C2, a known SND1 partner, and was thus RISC-independent. Identification of target mRNA species and interacting partners of Argonaute proteins might provide us with further insights into more precise roles of Argonaute proteins in colonic carcinogenesis [31].

## Conclusions

In conclusion, this immunohistochemical study of tissue microarray with 75 specimens from Chinese colon cancer and 75 specimens from adjacent non-tumorous tissue provided the first evidence that EIF2C1-4 and PIWIL1-4 were highly expressed in tumorous tissue relative to non-tumorous tissue. The expression of EIF2C2-4 and PIWIL4 appeared significantly increased in advanced tumors with distant metastasis, suggesting it may promote tumor invasion. Furthermore, EIF2C1 and PIWIL2 might represent novel colon cancer markers with early diagnostic significance. Taking all the information together, we postulate that at least some members of the human Argonaute family may be involved in the development and progression of colon cancer. Further study will be required to elucidate the exact biological function of each member of Argonaute family in human colon cancer.

## Competing interests

The authors declare that they have no competing interests.

## Authors' contributions

LL prepared tumor tissue arrays block, carried out the immunohistochemical staining, performed the statistical analysis, and drafted the manuscript. CHY participated in design and preparation of antibodies, and collected the human tissue and clinicopathological data. HJG reviewed the slides, evaluated the results of immunohistochemical staining and corrected the manuscript. YML designed and conducted the study. All authors read and approved the final manuscript.

## Pre-publication history

The pre-publication history for this paper can be accessed here:

http://www.biomedcentral.com/1471-2407/10/38/prepub
